# Efficacy and safety of Guhong injection for coronary heart disease: A protocol for systematic review and meta-analysis

**DOI:** 10.1097/MD.0000000000028447

**Published:** 2021-12-30

**Authors:** Sihua Che, Meiling Wang, Le Liu, Shumao Zhang, Guijun Shi

**Affiliations:** a School of Traditional Chinese Medicine, Changchun University of Traditional Chinese Medicine, Changchun, China; b Rehabilitation Hospital of Jilin Province Disabled People's Federation, China; c Changchun Chinese Medicine Hospital, Changchun, China.

**Keywords:** coronary heart disease, Guhong injection, meta-analysis, protocol

## Abstract

**Background:**

Coronary heart disease (CHD) is considered one of the major causes of morbidity and mortality worldwide, posing a serious threat to public health. Current therapeutic approaches for CHD mainly focus on drug therapy, coronary artery bypass grafting, and percutaneous coronary intervention. However, there still exist some problems including drug side effects, adverse cardiac events after percutaneous coronary intervention. Guhong injection is a compound preparation of traditional Chinese medicine and western medicine. Several clinical studies have shown that Guhong injection can effectively relieve the clinical symptoms of CHD patients and improve clinical efficacy. However, there is no systematic review to evaluate the effectiveness and safety of Guhong injection in treating CHD. Therefore, in this study we will plan to systematic review to evaluate the effectiveness and safety of Guhong injection for CHD, providing a strong evidence-based medical reference for clinical use.

**Methods:**

The database search includes EMBASE, PubMed, Cochrane Library, Web of Science, China National Knowledge Infrastructure, WanFang Database, Chinese Biomedical Database, Chinese Scientific Journal Database. The retrieval time was from their inception to November 30, 2021. The main outcome indicators include the frequency, severity, and duration of angina pectoris attacks, electrocardiogram changes, and dose of nitroglycerin. The analysis software uses RevMan 5.3.

**Results:**

By collecting the existing evidence, the results of this study will systematically evaluate the effects of Guhong injection in the treatment of CHD.

**Conclusion:**

The results of this study will provide evidence for the efficacy and safety of Guhong injection in the treatment of CHD.

**INPLASY Registration number:**

INPLASY2021120032.

## 1 Introduction

Coronary heart disease (CHD) is mainly the pathological change of coronary atherosclerotic plaque, which results in lumen stenosis or spasm, and eventually leads to myocardial ischemia, hypoxia, and even necrosis.[^1^,^2^] Various risk factors are closely associated with the occurrence of CHD, such as age, smoking, hypertension, diabetes, hyperlipidemia, anxiety, etc.^[3]^ CHD is considered one of the major causes of morbidity and mortality worldwide, posing a serious threat to public health.^[4]^ Current therapeutic approaches for CHD mainly focus on drug therapy, coronary artery bypass grafting, and percutaneous coronary intervention. However, there still exist some problems including drug side effects, adverse cardiac events after percutaneous coronary intervention (i.e., restenosis, stent thrombosis, and ischemia-reperfusion injury). As an important part of complementary and alternative medicine, traditional Chinese medicine has been widely used in clinical practice for thousands of years in China. In recent years, traditional Chinese medicine injections have achieved good results in the treatment of CHD.

Guhong injection is a compound preparation of traditional Chinese medicine and western medicine, which consists of acetyl glutamine and safflower extract. Modern pharmacological research has found that it has the effects of expanding coronary arteries, improving microcirculation, and reducing myocardial oxygen consumption.[^5^,^6^] Several clinical studies have shown that Guhong injection can effectively relieve the clinical symptoms of CHD patients and improve clinical efficacy.[^7^,^8^,^9^] However, there is no systematic review to evaluate the effectiveness and safety of Guhong injection in treating CHD. Therefore, in this study we will plan to systematically review and carry out an analysis to evaluate the effectiveness and safety of Guhong injection in the treatment of CHD, providing a strong evidence-based medical reference for clinical use.

## 2 Methods

### 2.1 Protocol and registration

This protocol will be conducted under the preferred reporting items for systematic reviews and meta-analyses protocols (PRISMA-P) guidelines.^[10]^ Furthermore, the study has been registered on INPLASY (https://inplasy.com/), registration number: INPLASY2021120032.

### 2.2 Type of study

The study will incorporate all randomized controlled trials that evaluate the efficacy of Guhong injection for the treatment of CHD, with no limit to language, publication, time, or blinding.

### 2.3 Type of participant

The patients who meet the diagnostic criteria of CHD will be included, including stable angina pectoris and unstable angina. There are no restrictions on sex, age, and race.

### 2.4 Intervention

The intervention method of the control group was conventional treatment, such as b-blockers, clopidogrel, statins, aspirin, etc. The treatment group was treated with Guhong injection combined with conventional treatment. There are no limitations on dosage, frequency, and duration. Additionally, we will exclude studies that combined with other herbal formulas, acupuncture, or moxibustion.

### 2.5 Outcomes

The primary outcomes will include the frequency, severity, and duration of angina pectoris attacks, electrocardiogram changes, and dose of nitroglycerin. The secondary outcomes will include blood lipid profile (triglyceride, total cholesterol, low-density lipoprotein cholesterol, and high-density lipoprotein cholesterol), Traditional Chinese medicine syndrome score scale, and adverse events.

### 2.6 Search strategy

We will perform a comprehensive systematic literature search in the following electronic databases: EMBASE, PubMed, Cochrane Library, Web of Science, China National Knowledge Infrastructure, WanFang Database, Chinese Biomedical Database, Chinese Scientific Journal Database. The retrieval time was from their inception to November 30, 2021. Using the following search terms: “Guhong injection,” “coronary heart disease,” “angina pectoris,” and “randomized controlled trials.” Illustrated by the case of PubMed, the detailed search strategy is shown in Table 1, which will make appropriate adjustments according to the specific database. Moreover, references of retrieved articles were also manually retrieved to obtain additional relevant studies.

**Table 1:** Search strategy of the PubMed.

Number	Search terms
#1	Coronary Disease[MeSH]
#2	Coronary Diseases[Title/Abstract] OR Disease, Coronary[Title/Abstract] OR Diseases, Coronary[Title/Abstract] OR Coronary Heart Disease[Title/Abstract] OR Coronary Heart Diseases[Title/Abstract] OR Disease, Coronary Heart[Title/Abstract] OR Diseases, Coronary Heart[Title/Abstract] OR Heart Disease, Coronary[Title/Abstract] OR Heart Diseases, Coronary[Title/Abstract]
#3	Angina Pectoris[MeSH]
#4	Stenocardia[Title/Abstract] OR Stenocardias[Title/Abstract] OR Angor Pectoris[Title/Abstract]
#5	#1 OR #2 OR #3 OR #4
#6	Injections[MeSH]
#7	Injection[title/abstract] OR Injectables[title/abstract] OR Injectable[title/abstract]
#8	#6 OR #7
#9	Guhong[MeSH]
#10	Guhong[title/abstract]
#11	#9 OR #10
#12	Randomized controlled trial [Publication Type] OR Controlled clinical trial[Publication Type]
#13	Randomized[Title/Abstract] OR randomly[Title/Abstract]
#14	#12 OR #13
#15	#5 AND #8 AND #11 AND #14

### 2.7 Data collection and analysis

#### 2.7.1 Selection of studies

**Figure 1:**
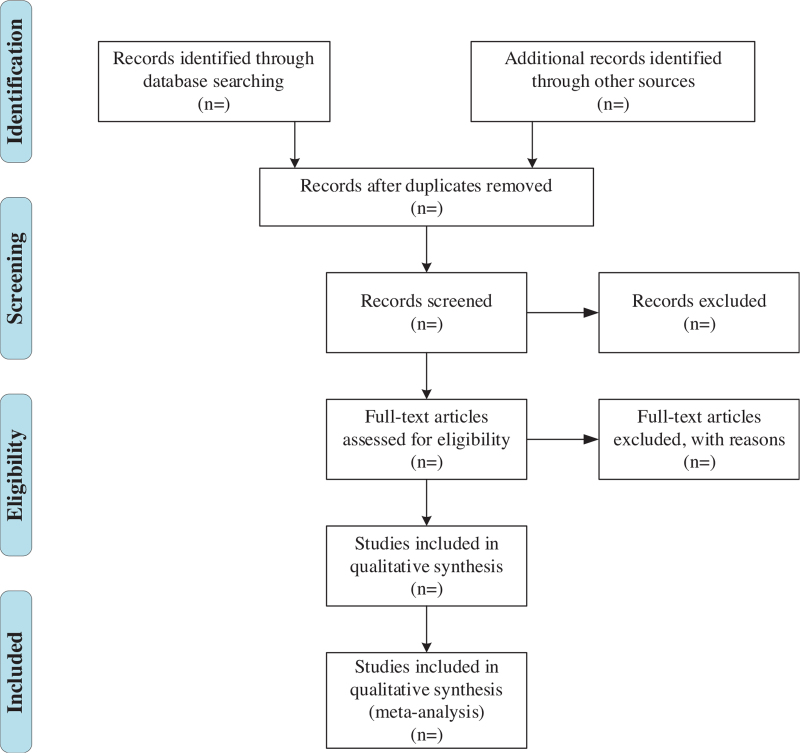
PRISMA flow diagram of the study selection process. PRISMA = preferred reporting items for systematic reviews and meta-analyses.

All retrieved literature that meets the requirements will be imported into Endnote X9 software (Camelot UK Bidco Limited, London, United Kingdom). Firstly, 2 researchers (SC and LL) will independently screen the titles and abstracts to identify the potentially eligible articles. Secondly, disqualified literature will be removed based on the inclusion criteria through downloading and reviewing the full text. The reasons for exclusion should be recorded at the same time. Finally, the final eligible articles selected will be carefully cross-checked by 2 researchers. Any disagreements will be mediated via a third investigator (MW) to reach a consensus. The process of studies selection according to the PRISMA flow chart is illustrated in Fig. 1.

#### 2.7.2 Data extraction and management

Two researchers (SC and SZ) will independently extract data according to a standard data extraction form by using Excel2013 software. The details of the form are as follows: basic information of studies (authors, title, and publication year), participants characteristics (age, gender, numbers, and course of disease), interventions, outcome indicators, adverse events, etc. If there is any incomplete information in the study, we will contact the corresponding author. All disagreements will be resolved by consulting a third researcher (GS).

#### 2.7.3 Risk of bias assessment

The risk of bias will be assessed independently by 2 researchers (SHC and LL) according to the tool of Cochrane Collaboration.^[11]^ The assessment items mainly contain random sequence generation, allocation concealment, blinding of participants and personnel, blinding of outcome assessors, incomplete outcome data, selective outcome reporting, and other sources of bias. Each item will be evaluated into 3 grades: “low risk,” “high risk,” and “unclear risk.” If necessary, a third researcher (MLW) will be involved to resolve the disagreements.

#### 2.7.4 Data synthesis and statistical analysis

Data synthesis and statistical analysis will be performed using the RevMan 5.3 (The Nordic Cochrane Centre, The Cochrane Collaboration, Copenhagen, Denmark) software provided by Cochrane Collaboration. The standard mean difference or mean difference with 95% confidence interval will be used to calculate the continuous data, while the dichotomous data will be measured by the rate ratio or odds ratio with 95% confidence interval. For the assessment of heterogeneity, the Chi-squared and *I* ^2^ test will be carried out. If there is no significant heterogeneity among studies (*I* ^2^ < 50%, *P* > .1), we will use a fixed-effect model, but a random-effects model will be employed if there exists heterogeneity (*I* ^2^ ≥ 50%, *P* < .1). Moreover, subgroup analysis and sensitivity analysis will be conducted to find the potential reasons for heterogeneity.

#### 2.7.5 Subgroup analysis

When there is significant heterogeneity among the studies, subgroup analysis will be performed according to the following factors: age, gender, types of CHD, the dosage of Guhong injection, and course of treatment.

#### 2.7.6 Sensitivity analysis

Sensitivity analysis will be conducted by eliminating included studies one by one and changing the statistical methods to assess the stability and reliability of analytical results.

#### 2.7.7 Publication bias

A funnel plot will be applied to examine the publication bias when there are >10 studies included and test for the symmetry of the funnel plot by using Egger tests.

#### 2.7.8 Grading the quality of evidence

The quality of evidence for the whole research will be assessed by the Grades of Recommendations Assessment, Development and Evaluation (GRADE) system,^[12]^ which was divided into 4 grades: “very low quality,” “low quality,” “medium quality,” and “high quality.”

#### 2.7.9 Ethics and dissemination

The approval of the ethics committee is not necessary, because that this article is a systematic review without involving the individual data of patients. Our results of work will disseminate in professional academic journals.

## 3 Discussions

CHD is the most common type of cardiovascular disease, which is mainly caused due to stenosis or obstruction of the coronary artery by atherosclerosis. Despite the fact that reduction in mortality of CHD over the past few decades, it is still the largest cause contributing to cardiovascular death worldwide.^[13]^ Although adherence to current guideline-recommended CHD therapy has improved overall survival rates, there still exist inevitable side effects. Therefore, it is particularly important to find a safe and effective treatment for CHD. In recent years, a large number of studies have shown that Guhong injection has definite effects in relieving the clinical syndrome of CHD patients.[^14^,^15^] However, the efficacy of Guhong injection in the treatment of CHD is still uncertain. Thus, our research will be the first systematic review to evaluate the effectiveness and safety of Guhong injection for CHD. It is expected to provide reliable evidence for the clinical application of Guhong injection to treat CHD.

## Author contributions

Literature retrieval and research will be completed by Sihua Che. Sihua Che wrote the first draft. Meiling Wang developed a search strategy. Le Liu and Shumao Zhang will conduct literature search and sorting. Sihua Che, Meiling Wang, and Le Liu will assess the risk of bias in the literature. Data analysis and article writing will be completed by Sihua Che and Meiling Wang. The corresponding author Guijun Shi is responsible for supervising all aspects of the review and controlling the quality of the research. All authors agreed to publish the plan.

**Conceptualization:** Sihua Che.

**Data curation:** Meiling Wang, Le Liu.

**Funding acquisition:** Guijun Shi.

**Investigation:** Shumao Zhang.

**Methodology:** Sihua Che.

**Project administration:** Guijun Shi.

**Resources:** Le Liu.

**Supervision:** Shumao Zhang.

**Validation:** Meiling Wang.

**Writing – original draft:** Sihua Che.

**Writing – review & editing:** Sihua Che.
